# When threats become catalysts: cognitive job crafting, work meaningfulness, and employee proactivity in high-insecurity contexts

**DOI:** 10.3389/fpsyg.2025.1513461

**Published:** 2025-04-11

**Authors:** Lidan Liu, Yuhan Su, Zhongjun Wang

**Affiliations:** ^1^School of Health Humanities, Hubei University of Chinese Medicine, Wuhan, Hubei, China; ^2^School of Psychology, Central China Normal University, Wuhan, Hubei, China

**Keywords:** cognitive job crafting, employee innovation behavior, job insecurity, organizational citizenship behavior, work meaningfulness

## Abstract

Drawing on Conservation of Resources (COR) theory, this study examined how cognitive job crafting stimulated employee innovation behavior and organizational citizenship behavior (OCB) through work meaningfulness, and how job insecurity moderated these effects. Using a multi-source, employee-supervisor paired design, data were collected from 512 Chinese participants across diverse industries (e.g., manufacturing, technology, healthcare). Employees self-reported cognitive job crafting, work meaningfulness, and job insecurity, while supervisors evaluated innovation behavior and OCB. A moderated mediation analysis revealed that cognitive job crafting significantly enhanced work meaningfulness, which in turn promoted both innovation behavior and OCB. Job insecurity amplified these indirect effects: under high insecurity, the mediating role of work meaningfulness was stronger. These findings highlighted cognitive job crafting’s unique role as a low-resource strategy to counter uncertainty. By bridging COR theory with job crafting research, this study advanced a resource-based perspective on employee adaptability in unstable environments.

## Introduction

Traditional job design theories posit that organizations structure tasks, skills, and relationships to shape employee attitudes (Hackman and Oldham, 1976; Morgeson and Humphrey, 2006). However, this top-down approach struggles to adapt to dynamic work environments. Employees increasingly engage in *job crafting*—an employee-initiated, individualized, bottom-up approach to job redesign (Wrzesniewski and Dutton, 2001). Job crafting is regarded as a crucial means for employees to autonomously pursue meaningfulness, cultivate job identity, and enhance person-environment fit, especially in uncertain settings (Tims and Bakker, 2010; Wrzesniewski and Dutton, 2001). A multitude of studies have verified the positive impacts of job crafting on work-related outcomes such as job satisfaction, work engagement, and task performance (Bohnlein and Baum, 2020; Lichtenthaler and Fischbach, 2019; Rudolph et al., 2017).

Despite job crafting’s diverse forms, prior studies often treat these crafting strategies homogenously (Demerouti et al., 2017) or prioritize behavioral job crafting (e.g., Bakker et al., 2012; Tims et al., 2016). Cognitive job crafting—mentally reframing work perceptions—remains understudied, with limited quantitative evidence (Lazazzara et al., 2020; Wang et al., 2023). Contextual constraints (e.g., rigid rules, limited autonomy) often hinder behavioral job crafting (e.g., task crafting, relational crafting), especially in lower-level roles (Berg et al., 2010; Lipscomb et al., 2006). However, cognitive job crafting thrives in such environments by allowing employees to reinterpret unchangeable tasks and relations (Berg et al., 2013; Zhang and Parker, 2019). When confronted with unchangeable limitations and constraints, modifying one’s perception of the job can be an effective crafting strategy (Berg et al., 2013; Fuller and Unwin, 2017; Wang et al., 2023; Zhang and Parker, 2019). While Wrzesniewski and Dutton (2001) posited the benefits of cognitive job crafting, empirical validation remains scarce (Melo et al., 2021). This gap is critical because cognitive job crafting requires minimal resource investment, making it uniquely viable in unchangeable and constrained contexts (Berg et al., 2013; Zhang and Parker, 2019).

**Figure 1:**
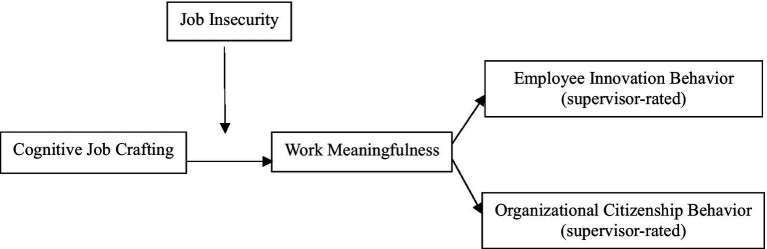
Overarching conceptual model.

Drawing on the Conservation of Resources (COR) theory (Hobfoll, 1989), we argue that cognitive job crafting serves as a strategic resource investment. By reshaping how employees perceive their work (e.g., aligning tasks with personal values), cognitive job crafting may enhance *work meaningfulness*—a work-related psychological resource that could fuel proactive behaviors like innovation and organizational citizenship behavior (OCB) (Steger et al., 2012; Allan et al., 2019). COR theory further suggests that individuals under resource threats (e.g., job insecurity) prioritize resource acquisition (Hobfoll et al., 2018). This implies cognitive job crafting’s effects may intensify when employees face high threats, as they strive to offset potential losses by deriving meaning from work. *Job insecurity*—a pervasive stressor in uncertain economies (De Witte, 1999)—provides a critical context to test this proposition. While insecurity typically depletes motivation (Jiang et al., 2021), the “gain paradox principle” of COR theory posits that threatened employees may paradoxically invest more in crafting to secure resources (Hobfoll et al., 2018). For instance, fearing job loss could drive employees to reframe their roles as meaningful, thereby stimulating innovation behavior and OCB to demonstrate value. This moderating role of insecurity remains unexplored, limiting practical strategies for organizations navigating volatility. Figure 1 shows our hypothetical model.

This study contributes to job crafting and COR literature in several ways. First, it answers scholars’ calls for more research on the role of specific job crafting strategies, especially cognitive job crafting (Geldenhuys et al., 2021; Slemp and Vella-Brodrick, 2013; Wang et al., 2023). Second, it links cognitive job crafting to innovation behavior and OCB through work meaningfulness, expanding outcomes beyond traditional attitudinal measures. Third, it reveals job insecurity as a boundary condition, offering insights into how organizations can leverage cognitive interventions amid uncertainty. By integrating these perspectives, we provide a nuanced framework for fostering employee proactivity in high-stress contexts. Practically, this study informs organizations on leveraging cognitive job crafting as a low-cost intervention to mitigate job insecurity’s adverse effects, such as designing role-reframing workshops or integrating crafting goals into leadership training.

## Theory and hypotheses

### Cognitive job crafting and COR theory

Job crafting, conceptualized as a bottom-up, employee-driven process of redefining work boundaries and perceptions (Lichtenthaler and Fischbach, 2019), has been theorized through two primary lenses. The *role-based perspective* (Wrzesniewski and Dutton, 2001) posits that employees proactively modify task (e.g., altering work content), relational (e.g., reshaping interactions), or cognitive boundaries (e.g., reframing work meaning), whereas the *resource-based perspective* (Tims et al., 2012) emphasizes aligning job demands and resources with personal needs (e.g., optimizing skill utilization). This study adopts Wrzesniewski and Dutton’s (2001) framework for three interrelated reasons. First, cognitive job crafting occupies a central role in their model, a focus that remains underemphasized in later frameworks prioritizing behavioral adaptations (Zhang and Parker, 2019). Second, its explicit distinction of cognitive crafting as a standalone strategy aligns with Conservation of Resources (COR) theory’s emphasis on psychological resource dynamics, enabling a nuanced analysis of meaning-making processes. Finally, the role-based perspective uniquely addresses proactive agency in constrained environments, contrasting with Tims et al.’s (2012) focus on balancing tangible job characteristics. By integrating these theoretical threads, we advance a context-sensitive understanding of cognitive job crafting’s role in fostering resilience under instability.

Cognitive job crafting reflects employees’ proactive efforts to mentally reframe work perceptions (e.g., linking tasks to personal values or redefining roles holistically) when behavioral adjustments are constrained (Wrzesniewski and Dutton, 2001). Employees can engage in cognitive job crafting in multiple ways. Berg et al. (2013) have putted forward three specific methods for cognitive job crafting: (1) Expanding perceptions, which involves viewing the job holistically rather than as a collection of fragmented tasks, thus broadening the meaning and value of the job; (2) Focusing perceptions, which involves mentally dividing the job into meaningful and meaningless parts, directing more attention to the meaningful aspects of the work; (3) Linking perceptions, which involves creating a psychological connection between the job and one’s personal interests, passions, or values. While behavioral job crafting requires tangible changes to tasks or relationships, cognitive job crafting operates through psychological reframing, making it more feasible in rigid work environments (Berg et al., 2013). This distinction highlights its unique role in enhancing work meaningfulness when behavioral adjustments are constrained (Wang et al., 2023). Unlike task or relational crafting, cognitive strategies demand minimal external resources, enabling employees to sustain motivation in rigid environments (Zhang and Parker, 2019). For example, a factory worker might reinterpret repetitive tasks as contributing to community well-being, thereby enhancing psychological resilience.

The COR theory (Hobfoll, 1989) serves as the fundamental theoretical underpinning for this study. From the COR perspective, job crafting (including cognitive job crafting) is essentially a proactive strategy for managing or investing work resources. Prior research has demonstrated that job crafting can assist employees in acquiring key resources, such as positive affect (Slemp et al., 2015) and self-efficacy (van den Heuvel et al., 2015). Recently, cognitive job crafting has been referred to as “resource crafting-metacognition” (Bruning and Campion, 2018) or “cognitive resource crafting” (Zhang and Parker, 2019). In line with numerous previous studies, work meaningfulness is regarded as a work resource since it plays a pivotal role in facilitating the attainment of other valuable outcomes such as job satisfaction, job performance, and overall well-being (Allan et al., 2019). COR posits that individuals facing resource threats (e.g., job insecurity) prioritize actions to offset potential losses (Hobfoll et al., 2018). Cognitive job crafting aligns with this logic: by reframing perceptions, employees convert constrained roles into meaningful experiences, fostering intrinsic motivation for innovation and citizenship behaviors. Notably, under high insecurity, the urgency to secure resources amplifies cognitive job crafting’s utility, as employees strive to mitigate instability through meaning-making (Hobfoll et al., 2018).

### Cognitive job crafting and employee innovation behavior

Cognitive job crafting involves mentally reframing work perceptions (e.g., viewing tasks as aligned with personal values or societal contributions) to enhance work meaningfulness (Wrzesniewski and Dutton, 2001). According to COR theory (Hobfoll, 1989), we argue that this cognitive adjustment represents a resource investment that fuels intrinsic motivation—a critical driver of innovation (Amabile, 1996). Employee innovation behavior is defined as the proactive actions taken by employees to introduce novel ideas, advocate new technologies, enhance existing processes or products, and effectively implement these innovations within the workplace (Scott and Bruce, 1994). Employees who perceive their work as meaningful are more likely to engage in exploratory behaviors. For instance, they may propose novel ideas or seek to improve processes. This occurs because meaningfulness lowers their perceived risks of innovation (Steger et al., 2012). Berg et al. (2013) found that through cognitive job crafting, employees were better equipped to handle complex work tasks and put forward innovative solutions. Evidence supports this link: Zhang and Parker (2019) proposed that cognitive crafting enhances self-efficacy, enabling employees to tackle complex tasks creatively, while Lazazzara et al. (2020) demonstrated its direct impact on innovative capability in rigid work environments. Therefore, we put forward the following hypothesis:

*Hypothesis 1*: Cognitive job crafting is positively related to employee innovation behavior.

### Cognitive job crafting and organizational citizenship behavior

Building on COR theory’s emphasis on resource acquisition, we now turn to the relationship between cognitive job crafting and OCB. Unlike innovation behavior, which focuses on proactive change, OCB reflects voluntary contributions to organizational welfare. OCB refers to voluntary actions that benefit the organization beyond formal job duties, such as helping colleagues and proposing efficiency improvements (Organ, 1988; Podsakoff et al., 2000). By redefining work roles as socially or organizationally significant, cognitive job crafting fosters a sense of responsibility beyond formal duties (Berg et al., 2013). COR theory explains this as resource spillover: meaningful work (a work resource) motivates employees to invest additional resources in prosocial actions like helping colleagues or volunteering for extra tasks (Hobfoll, 1989). Existing research has established a significant positive correlation between work meaningfulness and OCB (Steger et al., 2012). The study by Lazazzara et al. (2020) revealed that cognitive job crafting not only improves employees’ job satisfaction and well-being but also stimulates them to engage in more prosocial behaviors, which are frequently manifested as OCB. Therefore, we propose the following hypothesis:

*Hypothesis 2*: Cognitive job crafting is positively related to organizational citizenship behavior.

### The mediating role of work meaningfulness

Work meaningfulness is defined as the significance of work to individuals, encompassing their sense of belonging, role fit, and positive work experiences (Bailey et al., 2019; Steger et al., 2012). Cognitive job crafting could directly enhance work meaningfulness by enabling employees to reconstruct fragmented tasks into coherent, purposeful narratives (Letona-Ibañez et al., 2021; Geldenhuys et al., 2021). COR theory posits that meaningfulness acts as a “resource caravan” (Hobfoll et al., 2018), channeling psychological energy into innovation. Work meaningfulness usually cultivates intrinsic motivation, making employees more eager to go beyond routine work, put forward innovative suggestions, and exhibit higher creativity, thus engaging in exploratory behavior (Amabile and Pratt, 2016). For instance, Grant and Berry (2011) found that meaningful work amplifies creativity by aligning employees’ goals with organizational missions. Conversely, Tu and Lu (2013) showed that low meaningfulness leads to disengagement from innovative efforts. This mediation is further validated by studies linking cognitive job crafting to innovation through meaning-making (Lazazzara et al., 2020). Therefore, we propose the following hypothesis:

*Hypothesis 3*: Work meaningfulness mediates the relationship between cognitive job crafting and innovation behavior.

Work meaningfulness cultivates intrinsic motivation to contribute to organizational welfare (Spreitzer et al., 2005). Employees who cognitively reframe their roles as impactful are more likely to engage in OCB, as meaningfulness strengthens their identification with organizational goals (Wrzesniewski et al., 2013). For example, Geldenhuys et al. (2014) found that meaningful work predicts OCB through enhanced engagement, while Steger et al. (2012) highlighted its role in fostering prosocial values. COR theory reinforces this by framing meaningfulness as a resource that expands employees’ capacity for discretionary efforts (Chen et al., 2015). Empirical studies consistently demonstrate that work meaningfulness predicts OCB. For instance, Allan et al. (2019) found that meaningful work fosters prosocial behaviors, while Geldenhuys et al. (2021) linked cognitive crafting to higher OCB through enhanced meaningfulness. Therefore, we propose the following hypothesis:

*Hypothesis 4*: Work meaningfulness mediates the relationship between cognitive job crafting and organizational citizenship behavior.

### The moderating role of job insecurity

Job insecurity—a threat to resource stability (De Witte, 1999; Lee et al., 2018)—may intensify employees’ reliance on cognitive crafting to mitigate perceived losses. According to COR’s “gain paradox principle” (Hobfoll et al., 2018), individuals facing insecurity prioritize acquiring resources like meaningfulness to counterbalance external threats. For example, Jiang et al. (2021) found that insecure employees engage in more innovation to demonstrate indispensability. Similarly, Brzykcy et al. (2019) showed that resource-deprived individuals (e.g., those with disabilities) benefit disproportionately from cognitive interventions. Thus, high insecurity amplifies the mediation path: cognitive job crafting → meaningfulness → innovation. Thus, we propose the following hypothesis:

*Hypothesis 5*: Job insecurity moderates the indirect effect of cognitive job crafting on employee innovation behavior through work meaningfulness, such that the indirect effect is stronger under high level of job insecurity.

Under high insecurity, employees perceive OCB as a strategy to secure their positions (De Witte, 1999). Cognitive job crafting helps reframe instability as an opportunity to build social capital through prosocial behaviors (Piccoli et al., 2017). For instance, insecure employees may assist colleagues to strengthen team bonds, thereby reducing perceived replaceability. COR theory explains this as a “resource substitution” process: when job continuity is threatened, meaningfulness derived from cognitive job crafting becomes a substitute resource, driving OCB to compensate for instability (Hobfoll et al., 2018). Empirical studies have confirmed that insecure employees with high meaningfulness exhibit elevated OCB (Geldenhuys et al., 2021). Therefore, we propose the following hypothesis:

*Hypothesis 6*: Job insecurity moderates the indirect effect of cognitive job crafting on organizational citizenship behavior through work meaningfulness, such that the indirect effect is stronger under high level of job insecurity.

## Method

### Participants and procedures

This cross-sectional quantitative study employed a paired employee-supervisor design to test a moderated mediation model. Convenience and snowball sampling were used due to restricted organizational access. Data were collected via online surveys to minimize common method bias and mitigate social desirability bias (Podsakoff et al., 2003). A total of 80 undergraduate student volunteers, who were enrolled in a psychometrics course, were trained under the guidance of the researchers. These volunteers then introduced the study to potential participants from diverse industries and invited them to participate voluntarily. If the potential participants expressed their willingness to participate, they received a link to an online survey via smartphone applications. In the survey, employee participants provided demographic information and answered questions related to cognitive job crafting, work meaningfulness, and job insecurity. Upon completion of the survey, the participants received another link to a second online survey, which they were asked to forward to their immediate supervisor. In this survey, the supervisors were asked to voluntarily assess the employee’s innovation behavior and organizational citizenship behavior (OCB). Each employee and their supervisor were assigned a unique code in their respective questionnaires to match the responses from the employee and their supervisor.

Ethical approval for this study was obtained from the institutional review board (IRB) of the third author’s institution (Approval No. CCNU-IRB-202311034b). Participants provided informed consent via a digital form before accessing the survey, which outlined data confidentiality and voluntary participation. Supervisors received a separate consent form to ensure transparency. Each participant who successfully completed the surveys (both employees and their supervisors) received a monetary reward of 8 RMB. Each student volunteer who successfully recruited a participant-supervisor pair and ensured the completion of the surveys received a 6 RMB reward, along with extra course credit in the psychometrics class. Five hundred twelve successfully matched valid questionnaires were collected. The basic demographic information of the participants is as follows: among the participants, there were 202 males (39.5%); 116 were unmarried (22.7%); Participants’ average age was approximately 37 years (*M* = 36.94, *SD* = 9.48). Participants were from diverse industries (25% manufacturing, 18% technology, 17% business, 12% healthcare, 10% education, and 18% others). In terms of job positions, 304 participants were general employees (59.4%), 107 were junior managers (20.9%), 75 were middle managers (14.6%), and 26 were senior managers (5.1%). As for the types of organizations, 165 participants were from state-owned enterprises (32.2%), 221 from private enterprises (43.2%), 19 from foreign enterprises (3.7%), and 107 from other types of organizations (20.9%).

### Measures

#### Cognitive job crafting (self-reported by employee)

*Cognitive job crafting* was measured using the 5-item cognitive crafting subscale from the job crafting scale developed by Slemp and Vella-Brodrick (2013). An example item is “Thinking about how your job gives your life purpose.” Respondents were asked to what extent they had engaged in the different cognitive crafting strategies with each item, ranging from 1 (*never*) to 5 (*very often*). The Cronbach’s alpha for this scale was 0.91.

#### Work meaningfulness (self-reported by employee)

*Work meaningfulness* was assessed using the Steger et al. (2012) 10-item, three-dimensional scale, including positive meaning (*α* = 0.88) (4 items, e.g., “I understand how my work contributes to my life’s meaning”), meaning-making through work (*α* = 0.85) (3 items, e.g., “I have a good sense of what makes my job meaningful”) and greater good motivations (*α* = 0.82) (3 items, e.g., “My work helps me make sense of the world around me”). Participants indicated their agreement with each statement on a 5-point Likert scale, ranging from 1 (*strongly disagree*) to 5 (*strongly agree*). The Cronbach’s alpha for the total scale was 0.93.

#### Job insecurity (self-reported by employee)

Job insecurity was assessed using the Job Insecurity Scale developed by Hellgren et al. (1999), which includes 7 items across two dimensions: quantitative insecurity (*α* = 0.84) and qualitative insecurity (*α* = 0.88). Representative items include “I worry about losing my job in the future” and “My career prospects within this company are excellent” (reverse-coded). Responses were rated on a 5-point Likert scale, ranging from 1 (strongly disagree) to 5 (strongly agree). The Cronbach’s alpha for the total scale was 0.87.

#### Employee innovation behavior (supervisor-rated)

Employee innovation behavior was measured using the unidimensional scale developed by Scott and Bruce (1994), which assesses the extent of employee innovation in the workplace, including idea generation, seeking support for innovations, and implementing innovations. The scale consists of 6 items, such as “At work, he/she actively seeks out opportunities to implement new technologies, processes, or methods” and “He/she frequently comes up with creative ideas and innovative thoughts.” Responses were rated on a 5-point Likert scale, ranging from 1 (never) to 5 (always). The Cronbach’s alpha for the scale was 0.89.

#### Organizational citizenship behavior (supervisor-rated)

Organizational citizenship behavior (OCB) was measured using the two-dimensional scale developed by Bachrach et al. (2006), which assesses *helping behavior* (5 items; e.g., “He/she is willing to spend time helping colleagues who have work-related problems”) and *civic virtue* (5 items; e.g., “He/she provides constructive suggestions on how to improve organizational efficiency.”). Responses were recorded on a 5-point Likert scale ranging from 1 (*never*) to 5 (*always*). Both subscales demonstrated strong reliability, with Cronbach’s *α* values of 0.84 for helping behavior and 0.80 for civic virtue. The Cronbach’s alpha for the total scale was 0.83.

### Control variables

To avoid potential confounding effects, this study controlled for the participants’ gender (0 = male, 1 = female), age (in years), education (1 = elementary to 8 = doctoral), and position level (1 = general employee to 4 = senior manager), based on findings from previous studies (Hackett et al., 2018; Kim, 2018; Piff et al., 2010).

### Analytical approach

Data analysis proceeded in three sequential stages to rigorously test the hypothesized model. First, preprocessing addressed data quality: missing values (<5% of responses) were handled via full information maximum likelihood (FIML) estimation, preserving statistical power, while outliers (|*z*-scores| > 3.29) were winsorized to minimize distortion (Tabachnick and Fidell, 2019). Subsequently, measurement model validation was conducted using confirmatory factor analysis (CFA) in AMOS 22.0, where the hypothesized five-factor structure (cognitive job crafting, work meaningfulness, job insecurity, innovation behavior, OCB) was tested against alternative models. Model fit was evaluated using Hu and Bentler’s (1999) criteria: comparative fit index (CFI) ≥ 0.90 and root mean square error of approximation (RMSEA) ≤ 0.08. Finally, hypothesis testing employed hierarchical regression and Hayes (2013) PROCESS macro (Models 4 and 7) to examine mediation and moderated mediation effects. Control variables (gender, age, education, position) were mean-centered to reduce multicollinearity, and bootstrap confidence intervals (5,000 resamples) assessed effect stability. This multi-stage approach ensured robust validation of both measurement properties and theoretical relationships.

## Results

### Preliminary analyses

Before testing our hypotheses, confirmatory factor analyses (CFA) were conducted using AMOS 22.0 to assess the discriminant and convergent validity of all study variables. Considering the small sample size relative to the number of measured items, item parceling was used to reduce the number of indicators of each construct (Little et al., 2002). The five-factor model showed acceptable fit (CFI = 0.91, RMSEA = 0.05), aligning with thresholds for good fit (CFI ≥ 0.90, RMSEA ≤ 0.08; Hu and Bentler, 1999). Factor loadings ranged from 0.52 to 0.89, with average variance extracted (AVE) exceeding 0.50 for all constructs. Alternative models were also compared, indicating that the five-factor model fits the data considerably better than did any of the alternative models, thus supporting the discriminant validity of the measures (see Table 1). Table 2 presents descriptive statistics, composite reliabilities, and bivariate correlations for all the variables. As can be seen, the pattern of correlations was consistent with the proposed hypotheses (Figure 1).

**Table 1:** Confirmatory factor analysis results.

Model	*χ^2^*	*df*	χ^2^/*df*	GFI	CFI	RMSEA
One-factor model	6004.82	665	9.03	0.52	0.67	0.12
Two-factor model	4860.54	664	7.32	0.59	0.74	0.11
Three-factor model	4402.88	662	6.65	0.61	0.77	0.11
Four-factor model	3732.25	659	5.66	0.66	0.81	0.10
Five-factor model	2259.75	655	3.45	0.90	0.91	0.05

CJC, cognitive job crafting; WM, work meaningfulness; JI, job insecurity; IB, innovation behavior; OCB, organizational citizenship behavior; One-factor model = CJC+WM+JI+IB+OCB; Two-factor model = CJC+WM+JI, IB+OCB; Three-factor model = CJC+WM, JI, IB+OCB; Four-factor model = CJC, WM, JI, IB+OCB; Five-factor model = CJC, WM, JI, IB, OCB.

**Table 2:** Mean, standard deviations and correlations of study variables.

Variables	*M*	*SD*	1	2	3	4	5	6	7	8
1. Age	36.94	9.48								
2. Gender	1.61	0.49	0.04							
3. Education level	5.00	1.41	−0.02	−0.24**						
4. Position level	1.65	0.91	−0.18**	0.18**	0.08					
5. Cognitive job crafting	3.58	0.93	0.06	0.03	−0.18**	0.04				
6. Work meaningfulness	3.79	0.65	0.05	0.11*	−0.03	0.10*	0.47**			
7. Employee innovation behavior	3.86	0.81	−0.03	0.05	−0.08	0.18**	0.45**	0.42**		
8. Organizational citizenship behavior	4.08	0.57	0.08	0.13**	−0.15**	0.18**	0.47**	0.46**	0.46**	
9. Job insecurity	3.25	0.95	−0.03	−0.01	−0.12**	−0.06	0.35**	0.36**	0.30**	0.32**

*N* = 512; **p < 0.01, *p < 0.05.

### Test of hypotheses

We first used SPSS 25.0 to perform hierarchical regression analysis. As shown in Table 3, after controlling for gender, age, education level, and position level, cognitive job crafting was positively related to employee innovation behavior (*B* = 0.42, *SE* = 0.04, *p* < 0.01), supporting Hypothesis 1. Cognitive job crafting was also positively related to organizational citizenship behavior (*B* = 0.40, *SE* = 0.04, *p* < 0.01), supporting Hypothesis 2.

**Table 3:** The results of regression analysis.

Variables	Model 1:Innovation behavior		Model 2:OCB		Model 3:Work meaningfulness		Model 4:innovation behavior		Model 5:OCB	
	*B*	*SE*	*B*	*SE*	*B*	*SE*	*B*	*SE*	*B*	*SE*
Constant	1.48	0.21	2.38	0.14	3.32	0.12	3.90	0.22	1.69	0.14
Gender	0.08	0.06	−0.07	0.04	0.01	0.04	0.06	0.05	−0.08*	0.03
Age	0.01	0.01	0.01	0.01	0.01*	0.01	−0.01	0.01	0.01	0.01
Education level	0.02	0.02	−0.01	0.01	0.04**	0.01	−0.01	0.02	−0.03*	0.01
Position level	0.13**	0.03	0.09**	0.02	0.03	0.02	0.11**	0.03	0.08**	0.02
CJC	0.57**	0.03	0.40**	0.02	0.47**	0.02	0.27**	0.04	0.18**	0.03
JI					0.05*	0.02				
CJC × JI					0.09**	0.02				
Work meaningfulness							0.59**	0.05	0.44**	0.04
*R^2^*	0.44		0.48		0.56		0.55		0.59	
*F*	81.13**		92.50**		92.77**		101.93**		122.60**	

CJC, cognitive job crafting; JI, job insecurity; **p < 0.01, *p < 0.05.

Using the SPSS PROCESS macro Model 4 (Hayes, 2013), we tested the mediation effect while controlling for gender, age, education level, and position level. The bias-corrected percentile Bootstrap test indicated that the indirect effect of cognitive job crafting on innovation behavior through work meaningfulness was significant (Effect = 0.30, *Boot SE* = 0.04, 95% CI [0.23, 0.37]), supporting Hypothesis 3. Similarly, the indirect effect of cognitive job crafting on organizational citizenship behavior through work meaningfulness was significant (Effect = 0.23, *Boot SE* = 0.03, 95% CI [0.17, 0.28]), supporting Hypothesis 4.

**Figure 2:**
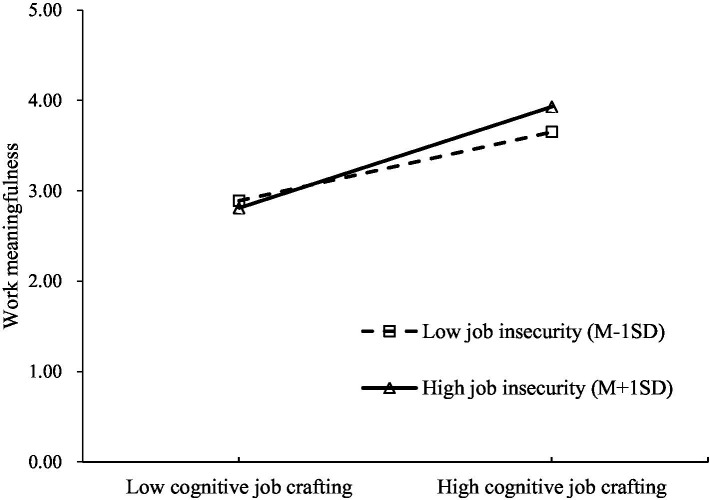
The relationship between cognitive job crafting and work meaningfulness moderated by job insecurity.

We used hierarchical regression analysis to test the moderating role of job insecurity, with the independent variable and the moderator variable being mean-centered. The results in Table 3 show that the interaction term between cognitive job crafting and job insecurity was positively related to work meaningfulness (*B* = 0.12, *SE* = 0.02, *p* < 0.01), indicating that job insecurity moderated the relationship between cognitive job crafting and work meaningfulness. As shown in the simple slope test results in Figure 2, compared to the low job insecurity condition (1*SD* below the mean, simple slope = 0.41, *t* = 9.96, *p* < 0.01), the positive relationship between cognitive job crafting and work meaningfulness was significantly stronger under high job insecurity (1*SD* above the mean, simple slope = 0.60, *t* = 15.79, *p* < 0.01). This suggests that employees in unstable contexts benefit more from cognitive job crafting.

We used the SPSS PROCESS macro Model 7 (Hayes, 2013; Preacher et al., 2007), controlling for gender, age, education level, and position level, to test the moderated mediation effect. As shown in Table 3, when job insecurity was low (i.e., 1*SD* below the mean), the indirect effect of work meaningfulness in the relationship between cognitive job crafting and employee innovation behavior was 0.23, with a 95% Bootstrap confidence interval that did not include zero, indicating a significant indirect effect. When job insecurity was high (i.e., 1*SD* above the mean), the indirect effect of work meaningfulness was 0.32, with a 95% Bootstrap confidence interval that also did not include zero, indicating a significant indirect effect. The difference in the indirect effects of cognitive job crafting at high and low levels of job insecurity was significant for innovation behavior (difference = 0.09, *SE* = 0.02, 95%CI [0.02, 0.09]), thus supporting Hypothesis 5 (Table 4).

**Table 4:** Conditional indirect effects of cognitive job crafting via work meaningfulness.

Conditon	Estimate	*SE*	95% Boot CI
Innovation behavior as outcome			
High job insecurity	0.32	0.04	[0.24, 0.40]
Low job insecurity	0.23	0.04	[0.16, 0.30]
Difference	0.09	0.02	[0.02, 0.09]
OCB as outcome			
High job insecurity	0.24	0.03	[0.18, 0.30]
Low job insecurity	0.17	0.03	[0.12, 0.23]
Difference	0.07	0.01	[0.02, 0.07]

Similarly, when job insecurity was low (i.e., 1*SD* below the mean), the indirect effect of work meaningfulness in the relationship between cognitive job crafting and OCB was 0.17, with a 95% Bootstrap confidence interval that did not include zero, indicating a significant indirect effect. When job insecurity was high (i.e., 1*SD* above the mean), the indirect effect of work meaningfulness was 0.24, with a 95% Bootstrap confidence interval that also did not include zero, indicating a significant indirect effect. The difference in the indirect effects of cognitive job crafting at high and low levels of job insecurity was significant for OCB (difference = 0.07, *SE* = 0.01, 95%CI [0.02, 0.07]), thus supporting Hypothesis 6.

## Discussion

This study investigated how cognitive job crafting was associated with employee innovation behavior and organizational citizenship behavior (OCB), with job insecurity moderating these effects. Analysis of multi-source data from 512 Chinese employees and their supervisors revealed two key findings. First, our findings demonstrated that cognitive job crafting was positively associated with employee innovation behavior and OCB through enhanced work meaningfulness. The positive relationship between cognitive job crafting and innovation behavior suggests that employees who reframe their work perceptions are more likely to generate novel ideas and explore novel solutions, as cognitive job crafting fosters intrinsic motivation to transcend routine tasks (Wrzesniewski and Dutton, 2001). Similarly, its link to OCB implies that meaningful work perceptions motivate employees to voluntarily support colleagues, even without formal incentives—a finding aligning with prosocial motivation theory (Grant and Berry, 2011; Organ, 2018). Second, our findings indicated that the mediating effects of work meaningfulness were stronger under high job insecurity. These results advance theoretical understanding of job crafting and resource management in volatile work contexts, while offering actionable strategies for organizations.

### Theoretical implications

First, this study addresses a critical gap in job crafting literature by systematically differentiating cognitive job crafting from its behavioral counterparts. Prior research often conflates diverse crafting strategies (e.g., task, relational, and cognitive) into a unified construct (Demerouti et al., 2017) or prioritizes behavioral forms due to their observable outcomes (Tims et al., 2016). Our findings demonstrate that cognitive job crafting operates through distinct psychological pathways: by reinterpreting work perceptions (e.g., aligning tasks with personal values or emphasizing broader purposes), employees cultivate work meaningfulness without altering tangible job characteristics (Berg et al., 2013; Wrzesniewski and Dutton, 2001). This is particularly significant in rigid work environments (e.g., manufacturing, healthcare) where behavioral adjustments are restricted by organizational protocols (Zhang and Parker, 2019). By empirically validating cognitive job crafting’s unique mechanisms, we respond to recent calls for dimensional specificity in job crafting research (Lazazzara et al., 2020; Tims et al., 2022; Wang et al., 2023).

Second, this research extends Conservation of Resources (COR) theory by positioning cognitive job crafting as a low-cost, high-impact resource investment strategy. While COR traditionally emphasizes tangible resource acquisition (Hobfoll, 1989), we show that psychological resources like work meaningfulness can act as “resource caravans” (Hobfoll et al., 2018), amplifying employees’ capacity to engage in innovation and citizenship behaviors. For instance, employees who cognitively reframe monotonous tasks as “building blocks for organizational success” not only preserve mental energy but also channel it into creative problem-solving (Amabile and Pratt, 2016). This aligns with COR’s principle of resource investment but introduces a novel perspective: cognitive job crafting allows individuals to optimize existing resources rather than solely pursuing new ones. Such findings deepen the theoretical dialog on resource management in constrained environments.

Third, we advance the job crafting literature by identifying job insecurity as a critical boundary condition. While insecurity is typically viewed as a stressor that depletes motivation (De Witte, 1999), our results align with COR’s “gain paradox” principle (Hobfoll et al., 2018), revealing that threatened employees paradoxically invest more in cognitive job crafting to secure psychological resources. This heightened sensitivity to resource gains amplifies the mediating role of work meaningfulness, as evidenced by the stronger indirect effects observed in our results. For example, under high insecurity, employees may reinterpret their roles as “indispensable crisis navigators,” thereby converting anxiety into proactive behaviors like innovation behavior and OCB to demonstrate value (Piccoli et al., 2017). This challenges conventional assumptions about insecurity’s uniformly negative effects and provides a nuanced framework for understanding adaptive responses to volatility.

### Practical implications

Organizations can leverage cognitive job crafting as a cost-effective tool to enhance resilience and performance. First, managers should train employees to reframe work perceptions through guided reflection exercises. For example, workshops could help retail workers view customer interactions as “relationship-building opportunities” rather than transactional duties. Second, in insecure contexts (e.g., mergers, economic downturns), leaders should emphasize meaning-making—such as highlighting how individual roles contribute to organizational stability—to mitigate anxiety and channel energy into innovation. Third, for organizations, we propose actionable interventions such as (1) training programs to reframe work perceptions (e.g., linking tasks to societal impact), and (2) integrating cognitive job crafting goals into performance feedback systems. Moreover, HR systems should recognize and reward both innovation behavior and OCB, reinforcing the value of cognitive job crafting.

### Limitations and future directions

Four limitations warrant attention. First, the cross-sectional design precludes causal claims. Future studies should employ longitudinal or experimental designs, such as tracking employees’ crafting behaviors before/after organizational changes. Second, due to challenges in accessing a random sample, convenience and snowball sampling were adopted in this study. While this approach allowed efficient data collection, it may limit generalizability. Moreover, overrepresentation of women (60.5%) and private-sector (43.2%) employees may also limit our study’s generalizability. Third, self-reported cognitive job crafting and meaningfulness may introduce bias. Triangulating with physiological data (e.g., heart rate variability during meaning-making tasks) could enhance validity. Fourth, while we focused on job insecurity, future studies could explore contextual factors (e.g., organizational climate, transformational leadership) and individual traits (e.g., proactivity) that may interact with cognitive job crafting. For example, proactive employees might leverage cognitive job crafting more effectively under supportive leadership (Zhang and Parker, 2019). Comparative studies across industries (e.g., stable vs. volatile sectors) could further generalize findings.

In conclusion, this study demonstrated that cognitive job crafting serves as a low-cost, high-impact strategy to foster innovation and OCB, particularly in insecure work environments. By integrating COR theory with job crafting research, we advance a resource-based perspective on employee adaptability. For organizations, these findings underscore the value of empowering employees to cognitively reframe their roles, even when structural changes are constrained.

## Data Availability

The original contributions presented in the study are included in the article/supplementary material, further inquiries can be directed to the corresponding author.
